# Concurrent vestibular activation and postural training recalibrate somatosensory, vestibular and gaze stabilization processes

**DOI:** 10.1371/journal.pone.0292200

**Published:** 2024-07-05

**Authors:** Kwadwo Osei Appiah-Kubi, Anne Galgon, Ryan Tierney, Richard Lauer, W. Geoffrey Wright

**Affiliations:** 1 Health & Rehabilitation Sciences Department, Temple University, Philadelphia, Pennsylvania, United States of America; 2 Physical Therapy Department, Clarkson University, Potsdam, New York, United States of America; 3 Physical Therapy Department, Saint Joseph’s University, Philadelphia, Pennsylvania, United States of America; Tokai University, JAPAN

## Abstract

Postural instability is a common symptom of vestibular dysfunction that impacts a person’s day-to-day activities. Vestibular rehabilitation is effective in decreasing dizziness, visual symptoms and improving postural control through several mechanisms including sensory reweighting of the vestibular, visual and somatosensory systems. As part of the sensory reweighting mechanisms, vestibular activation exercises with headshaking influence vestibular-ocular reflex (VOR). However, combining challenging vestibular and postural tasks to facilitate more effective rehabilitation outcomes is under-utilized. Understanding how and why this may work is unknown. The aim of the study was to assess sensory reweighting of postural control processing and VOR after concurrent vestibular activation and weight shift training (WST) in healthy young adults. Forty-two participants (18–35years) were randomly assigned into four groups: No training/control (CTL), a novel visual feedback WST coupled with a concurrent, rhythmic active horizontal or vertical headshake activity (HHS and VHS), or the same WST with no headshake (NHS). Training was performed for five days. All groups performed baseline- and post-assessments using the video head impulse test, sensory organization test, force platform rotations and electro-oculography. Significantly decreased horizontal eye movement variability in the HHS group compared to the other groups suggests improved gaze stabilization (*p* = .024). Significantly decreased horizontal VOR gain (*p* = .040) and somatosensory downweighting (*p* = .050) were found in the combined headshake groups (HHS and VHS) compared to the other two groups (NHS and CTL). The training also showed a significantly faster automatic postural response (*p* = .003) with improved flexibility (*p* = .010) in the headshake groups. The concurrent training influences oculomotor function and suggests improved gaze stabilization through vestibular recalibration due to adaptation and possibly habituation. The novel protocol could be modified into progressive functional activities that would incorporate gaze stabilization exercises. The findings may have implications for future development of vestibular rehabilitation protocols.

## Introduction

Postural stability is fundamental to performing activities of daily living and it relies on the central integration of somatosensory, visual and vestibular inputs through a process called sensory reweighting. During head and body movements, the vestibular system facilitates gaze stabilization and postural stability [1, 2]. Although it is relatively less weighted by the central nervous system and not routinely assessed clinically [3, 4], the vestibular system is crucial during eyes-closed quiet stance [5] and during activities that involve head and bodily movements. A dysfunctional vestibular system can lead to dizziness, vertigo and poor balance that can subsequently result in problems with walking and activities of daily living [3, 4].

Vestibular rehabilitation can be effective in decreasing dizziness and visual symptoms, and corrects for overdependence on the visual and somatosensory inputs [6]. It achieves these outcomes through the mechanisms of sensory reweighting, vestibular habituation, adaptation and/or substitution [6–9]. While vestibular *habituation* uses repeated exposures to provocative stimuli to decrease symptoms, *adaptation* regulates and adapts sensory neural processing to recalibrate the vestibulo-ocular reflex (VOR), and *substitution* aims to promote alternative strategies, such as, the use of saccades to replace the slow phase of VOR. Although the aforementioned motor learning processes have their general clinical trajectory to demonstrate improvement in postural function, the underlying neural processes modify the vestibular system by recalibrating the vestibular reflex pathways to improve postural stability [7–9]. For example, recalibrating the VOR or vestibulo-spinal reflex (VSR) gain up or down can lead to either an increased or decreased postural response in patients with hypofunction or hyperfunction, respectively.

Although vestibular rehabilitation can be effective, some patient groups may not improve [9]. A systematic review of randomized controlled trial studies (RCT) that used various vestibular rehabilitation protocols did not show significant effects on limits of stability (LOS), tandem position or vestibular symptoms in patients, although improvement in dynamic balance and activities of daily living was found [10]. Mitsutake et al. [11] employed vestibular rehabilitation through progressive gaze stabilization exercises (in sitting, standing and walking) and added balance exercises separately while actively rotating neck and trunk in post-stroke individuals. The experimental group significantly improved in gaze stabilization and dynamic gait index compared to an age-sex-matched cohort who performed only conventional rehabilitative intervention. Gaze stabilization exercises require rhythmic rotation of the head while fixating a target. Other studies have used gaze stabilization and habituation training exercises, including active rhythmic headshake and trunk flexion-extension exercises to reach targets. The studies show improvements in VOR gain, postural balance, dynamic visual acuity (DVA) and subjective dizziness scores [12–14]. These findings provide the basis for using postural training to augment vestibular rehabilitation. Gaze stabilization has also demonstrated improvement in postural balance and DVA in healthy adults [15, 16]. In a previous study, we used concurrent vestibular horizontal headshake activities and postural training to influence sensory weighting processes in healthy young adults [17]. The pre- and post-measures showed somatosensory upweighting, decreased center of pressure (COP) medio-lateral variability and an increase in multiscale entropy (MSE) sway velocity, all suggesting improved postural stability. This was one of the first studies to combine volitional headshake with postural activities performed simultaneously to optimize vestibular and postural function.

Concurrent vestibular headshake and weight shift training (concurrent HS-WST) capitalizes on the processes of vestibular recalibration (i.e., adaptation, a form of recalibration) and/or habituation to desensitize and consequently resolve vestibular symptoms and also to adapt sensory neural processing [18, 19]. In addition, the training uses sensory reweighting from the somatosensory system to modify spinal reflex responses [20, 21]. Demonstrating these outcomes in healthy young adults provides foundational data and evidence of safety and feasibility prior to embarking on large-scale trials with neurologic patients. Our aims were to assess the impact of concurrent HS-WST on VOR, eye movement behavior and sensory reweighting of postural control in healthy young adults and address limitations in our previous study [17]. We hypothesized a significant change would occur in these variables for those who undergo headshake training.

## Materials and methods

### Subjects

Healthy college students from Temple University were recruited for the study from April 10^th^ to September 27^th^, 2018. An a priori power analysis revealed that a minimum sample of 36 participants was required for a four-group comparison to detect a significant difference at alpha = 0.05 and 0.30 effect size at beta = 0.8 (G*Power, Version 3.0.10) [17, 22]. Participants were assessed for exclusion criteria with a short medical background form and a musculoskeletal examination. Participants were excluded if they had any evidence of concussion, vestibular, balance or oculomotor issues for the prior 6 months or any current musculoskeletal deficits including significant postural abnormalities, pain or limitations in neck range of motion. Forty-two young adults were randomized into four groups using a group by gender method [23]: 1) real-time visual feedback weight shift training (WST) coupled with an active horizontal headshake (HHS), 2) same WST with vertical headshake (VHS), 3) WST with no headshake (NHS) and 4) no training or no headshake (control, CTL) groups. This study was approved by the Temple University Institutional Review Board (Protocol# 23436; initial approval on 03/02/2018). All participants gave written informed consent, and all participants’ questions and concerns were answered by the researcher before the commencement of the study.

### Demographics

Participants’ mean age was 23.0±3.9years (18–35years) and mean height was 1.6±0.1m. No significant differences in the characteristics between groups at baseline were found (Table 1).

**Table 1:** Characteristics of participants (n = 42).

Variable	Horizontal Headshake (n = 11)	Vertical Headshake (n = 10)	No Headshake (n = 11)	Control (n = 10)	*p*
Age (yrs)	22.9±3.4	22.1±3.1	22.5±4.1	24.8±4.9	0.422
Height (m)	1.7±0.1	1.6±0.1	1.7±0.1	1.7±0.1	0.633
Weight (kg)	65.3±13.7	64.8±8.8	71.4±19.2	68.5±19.2	0.754
BMI (kg/m^2^)	23.3±3.2	24.1±3.0	25.0±4.9	23.9±5.2	0.833
Male (%)	5 (45%)	5 (50%)	5 (45%)	5 (50%)	0.994
Female (%)	6 (55%)	5 (50%)	6 (55%)	5 (50%)	

Age, height, weight and BMI (mean+standard deviation); sex (frequency).

### Outcome measures and assessment procedure

Two outcome assessments (i.e., baseline and post-training assessments) were performed on all the groups using four assessment tools:

1. VOR gain: This was assessed with a video head impulse test (vHIT; ICS, GN Otometrics, Taastrup, Denmark) system [24]. Participants were asked to sit 1.3m away from a target, keeping head, neck and trunk as still as possible while small, unpredictable horizontal head impulse rotations were passively delivered (sampling rate = 250Hz) [25]. Twenty head impulses (10 in each direction) were performed to assess the left and right horizontal semicircular canals (SCCs).
2. Static postural balance: The sensory organization test (SOT; NeuroCom^®^ Balance Master, Natus Medical Inc., Pleasanton, CA) required participants to stand upright as stably as possible for 20s under six different testing conditions: Condition 1 –eyes open (EO) on stable support (SS), Condition 2 –eyes closed (EC) on SS, Condition 3 –sway-referenced visual surround (SRv) on SS, Condition 4 –EO on sway-referenced support (SRs), Condition 5 –EC on SRs, Condition 6 –SRv on SRs [26]. The order of conditions was always 1–6, with each condition repeated 3 times, and the average used for analysis. This SOT procedure is in accordance with manufacturer specifications, which has been shown to be most reliable [27]. The sampling rate was 100Hz.
3. Dynamic postural balance: The ramp module of the NeuroCom^®^ Balance Master, (Natus Medical Inc., Pleasanton, CA) was used to assess the automatic postural responses of participants as they stood upright and were instructed to recover balance from unpredicted toes up and toes down perturbations simulating being push backwards and forwards, respectively. The angular velocity of the toe up or toe down rotations was ±50°/s with amplitude of 10° and 0.2s duration [28]. Six 5s trials (i.e., unpredictable three toes up followed by three toes down) were performed with random onset of 1s or 2s between each trial meant to elicit an automatic postural response.
4. Eye movement variables: The BlueGain electro-oculography (EOG) device (Cambridge Research Systems) [29–31] was used to assess the horizontal and vertical eye movements (sampling rate = 1kHz) during the toes up and toes down perturbations on the NeuroCom^®^ Balance Master. After skin preparation using isopropyl alcohol wipes, two sets of two “duck foot” surface gel electrodes (20 x 15mm, Ag/AgCl sensor; Ambu Neuroline 700, Denmark) were placed horizontally (near the lateral and medial canthi) and vertically (above the eyebrow and below the lower eyelid) around the right eye, with a reference electrode on the forehead. The calibration of the EOG device was performed synchronously during the vHIT system calibration.

### Training protocol

**Fig 1:**
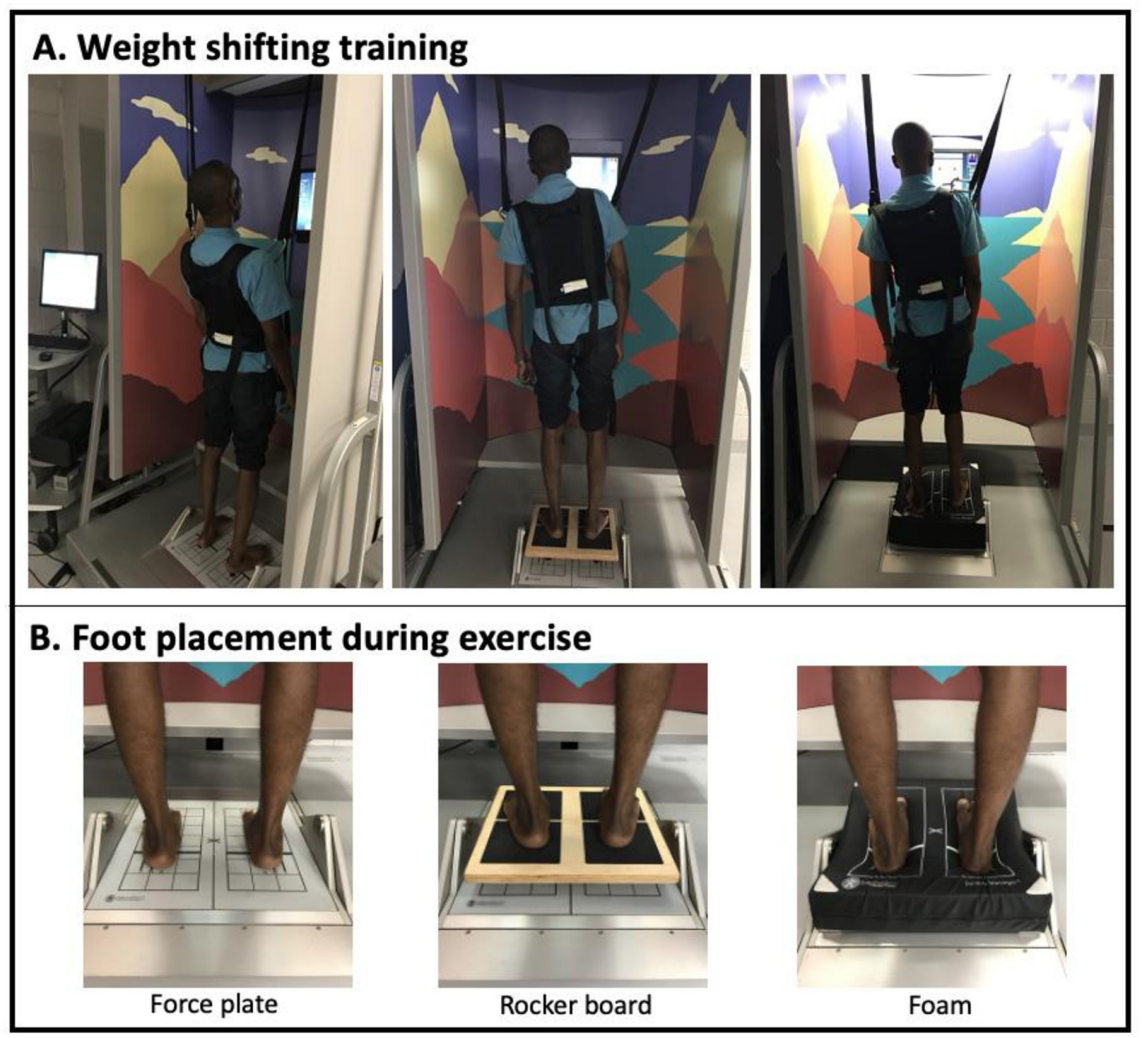
Training protocol. A. Weight shift exercises on force plate, rocker board and foam, with participant following directions on the screen in front of them. The screen provided visual feedback exercise targets of limits of stability (LOS), which switches randomly every 4 s. The LOS targets were arranged in a peripheral pattern where the participant leaned in a forward, backward or sideway direction. B. Foot placement during exercises.

The training groups (HHS, VHS and NHS) performed training once a day for four consecutive days, followed by a two-day (weekend) break and final session. Participants observed a demonstration of the first exercise prior to beginning the training. Participants performed all training using the NeuroCom^®^ WST protocol at level 3-forward and backward directions with 65%–70% LOS and level 5-forward direction with 85%–90% LOS (Fig 1).

Five exercises were used for each level. The exercises entailed participants leaning towards the specific direction related to the exercise number (i.e., forward, backward, diagonal or sideways) to reach a target that lights up randomly on the screen every 4s. Participants were instructed to quickly move their COM towards the highlighted target (while looking at the target) as soon as possible and maintain that position until the next target lit up. Details of the NeuroCom^®^ WST have been described elsewhere [17].

During the WST, participants in the HHS and VHS groups (headshake groups) were instructed to rhythmically rotate their heads horizontally or vertically approximately 30° at 80 to 120 beats/minute as the sessions progressed following the sound of an electronic metronome app (Best Metronome & Tuner). The headshake activities were performed in three out of five exercises for each level (except level 3 backwards), amounting to 6 out of the 15 exercises in each session [17]. The headshakes were not performed throughout all the 15 exercises in order to incorporate some rest breaks and avoid potential dizziness. There was a 15s rest period between each exercise and a 1min rest period between each level, totaling approximately 20min training per session. Throughout the training each participant in the training groups performed 1,125 COM shifts, 1,125 eye movements (volitional saccades), and, in addition, HS participants performed between 1380–1400 head movements. The number of COM shifts and volitional saccades were calculated as 5 training days x 15 exercises/day x 15 times that the target on the screen will change/exercise. Post-reassessments were performed 24h after the last training day. The control group only performed baseline and reassessments on day one and day six, respectively.

### Data management and analysis

The left and right horizontal VOR gains for each participant were obtained from the vHIT software output and expressed as a mean. A mean VOR gain was then calculated for each group. The SOT composite and equilibrium scores were computed by the NeuroCom^®^ Balance Master software. The SOT sensory ratios were computed using the equilibrium scores and are as follows: somatosensory ratio = condition 2/condition 1; visual ratio = condition 4/condition 1; vestibular ratio = condition 5/condition 1; and preference ratio = condition (3+6)/condition (2+5). The COP time series data of the SOT were transformed into COP sway area (using principal component analysis, PCA), sway velocity (using path length divided by total time), standard deviation of medio-lateral (ML SD) and antero-posterior (AP SD) sways, and multiscale entropy (MSE) sway velocity (m = 2; r = 0.2, tau = 10) using custom MATLAB codes (MathWorks, 2018). Data were filtered at 30Hz before the calculations. In addition, the first-200ms COP data of the toes up and down perturbation trials were considered for analysis, and sway variables similar to that of the SOT were analyzed. In a seated position with the head stable, the EOG horizontal and vertical signals were calibrated by asking participants to perform volitional saccades to two horizontal and vertical fixed points, respectively, to obtain calibration factors (deg/volts) for both directions. The signal of the actual trial (volts) during the toes up and toes down rotation was then multiplied by the respective calibration factor to obtain the raw signal in degrees. Data were filtered using a Butterworth lowpass filter of 2^nd^ order and at 30Hz cut-off frequency. Filtered data were detrended and the displacement (deg) was calculated for horizontal and vertical signals. The displacement time series was differentiated by time to derive velocity (deg/s). Means of the three trials were found from the rotation onset to the end of the 5s trials and from rotation onset to the next 200ms. Eye movement area (using PCA), velocity, horizontal eye movement and vertical eye movement were calculated. A between- and within-group repeated-measures (RM) ANOVA was used to analyze the five COP-SOT sway variables and SOT equilibrium scores for the six conditions (3 visual x 2 surface x 4 groups x 2 sessions). Also, RM ANOVA was used to analyze SOT composite scores, sensory ratios, VOR gains, COP toes up/down variables and eye movement variables (4 groups x 2 sessions). A significant omnibus ANOVA was followed-up with a planned comparison. Bonferroni post-hoc adjustments were used to adjust for multiple between-group comparisons. Violations of sphericity were checked by Mauchly’s test, but none were found. In cases where no significant difference was found between the groups, the headshake group data (HHS and VHS) were combined as one new group and compared to the NHS and CTL groups separately. This was deemed important since HHS and VHS involved active stimulation of the vestibular system, whereas NHS involved only passive or indirect vestibular stimulation (which occurred during the body lean activity). Hence this analysis helped to address the main aim of the study. All statistical analysis was conducted using SPSS software (v25.0; IBM Corp.) and significance set at α ≤ 0.05. A statistically significant group x session interaction would indicate a training effect.

## Results

### Horizontal VOR gain

**Fig 2:**
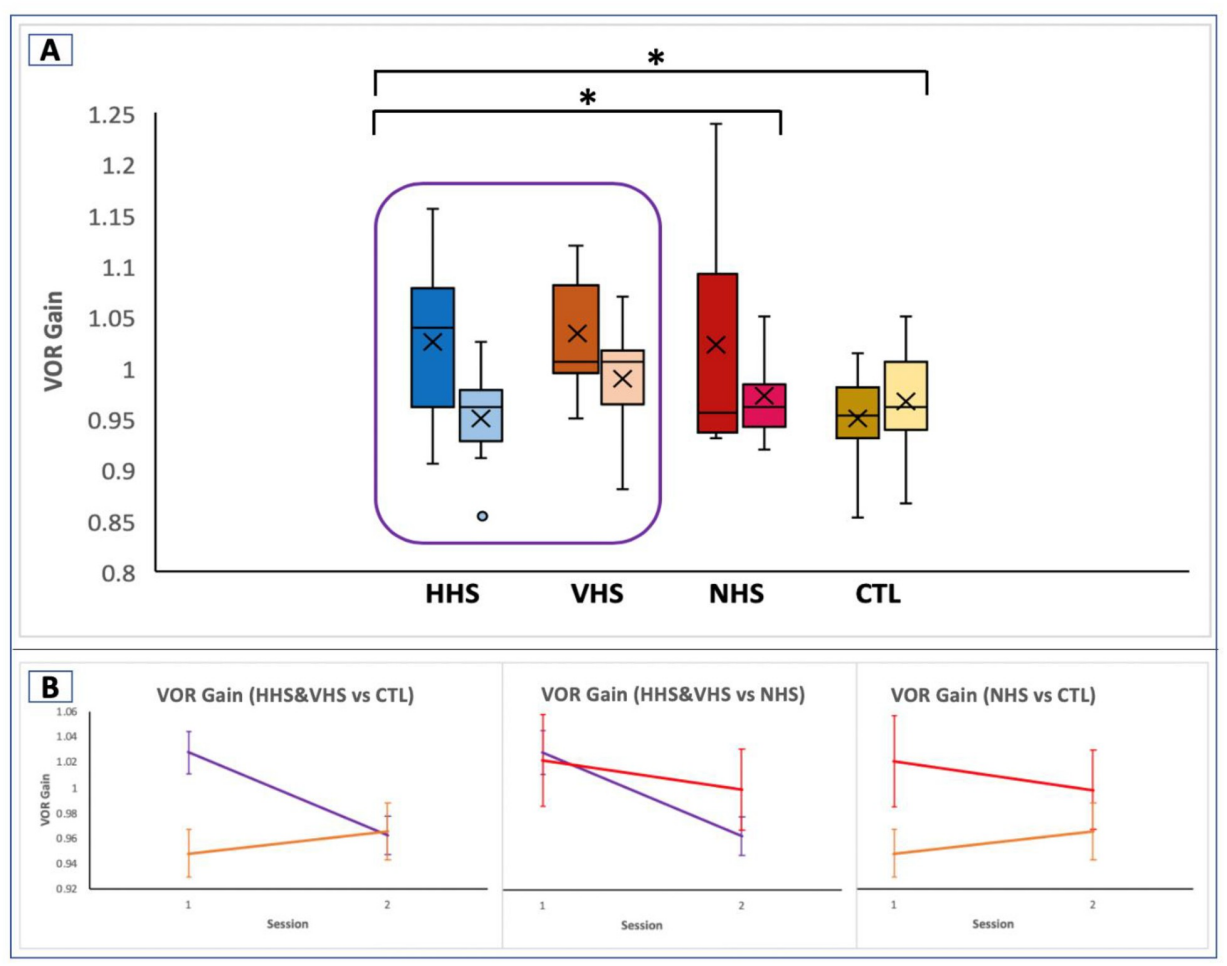
Effect of HS-WST on VOR gain. A. VOR gain for the 4 groups. Lighter colors indicate post-assessment. All three training groups showed a decrease in the VOR gain, with the controls, showing a slightly upward VOR gain. B. Planned comparison of the headshake groups against NHS and CTL. Purple = HHS + VHS, red = NHS, orange = CTL. Vertical bars indicate standard error bars. Headshake groups (HHS + VHS) showed significantly decreased VOR gain compared to CTL, but no significant change when headshake groups is compared to NHS and between NHS and CTL. * indicates significance at p≤0.05.

There was no statistically significant group x session interaction in horizontal VOR gain pre- to post-training. However, results showed a trending group x session (*F*_3,31_ = 2.53, *p* = .076) interaction pre- to post-training (Fig 2A).

Since the primary focus of the study was to investigate the effect of headshake (active vestibular stimulation) on oculomotor and postural outcomes, both headshake groups were pooled together (HHS+VHS) and compared to NHS and CTL separately. The analysis showed a statistically significant group x session interaction (*F*_2,32_ = 3.57, *p* = .040) due to training between the combined headshake groups versus NHS and between the combined headshake groups versus CTL when comparing pre- to post-training outcomes. Post hoc comparison showed a significant decrease in VOR gain in the headshake groups as compared to the controls (*F*_1,23_ = 8.33, *p* = .008). There were no differences between the headshake groups and NHS (*F*_1,25_ = 0.15, *p* = .704), and NHS and controls (*F*_1,16_ = 2.96, *p* = .105) (Fig 2B).

### SOT measures

The SOT equilibrium and composite scores (Table 2) and the SOT-COP sway measures (i.e., sway area, sway velocity, ML SD, AP SD and MSE velocity) did not show statistically significant training effects.

**Table 2:** SOT equilibrium and composite scores for each group.

Gp	Time-point	EO-SS C1	EC-SS C2	SRv-SS C3	EO-SRs C4	EC-SR C5	SRv-SRs C6	Composite
HHS	Pre	94.0±2.73	89.4±7.91	89.9±7.84	75.2±19.0	60.4±17.6	57.2±20.0	72.6±14.2
	Post	94.8±2.22	88.0±10.2	90.0±6.18	75.3±17.0	60.7±10.8	58.6±18.2	73.4±11.4
	Δ	0.80±0.51	-1.40±2.29	0.10±1.66	0.10±2.00	0.30±6.80	1.40±1.80	0.80±2.80
VHS	Pre	95.1±1.57	91.7±2.02	91.2±3.45	74.1±12.3	53.7±8.74	57.2±15.3	70.0±6.94
	Post	93.9±1.65	90.4±3.74	91.5±2.28	76.7±14.9	57.4±10.4	58.6±18.3	71.4±10.3
	Δ	-1.20±0.08	-1.30±1.72	0.30±1.17	2.60±2.60	3.70±1.66	1.40±3.00	1.40±3.36
NHS	Pre	94.5±1.59	89.6±3.88	89.0±3.56	82.3±13.2	69.1±8.51	65.5±11.2	78.1±6.88
	Post	94.8±1.00	88.7±5.00	90.1±3.32	76.3±17.1	67.6±13.3	62.4±18.0	76.5±10.5
	Δ	0.30±0.59	-0.90±1.12	1.10±0.24	-6.00±3.90	-1.50±4.79	-3.10±6.80	-1.60±3.62
CTL	Pre	94.7±1.36	91.6±3.46	91.6±4.12	82.4±9.42	64.9±8.86	60.9±9.94	77.2±5.50
	Post	93.8±1.64	92.7±1.62	92.3±1.73	83.5±7.23	69.6±8.00	63.5±8.67	79.5±4.91
	Δ	-0.90±0.28	1.10±1.84	0.70±2.39	1.10±2.19	4.70±0.86	2.60±1.27	2.30±0.59

HHS = Horizontal headshake, VHS = Vertical headshake, NHS = No headshake, CTL = Control, Gp = Group; EO = Eyes open, EC = Eyes closed, SS = Stable support, SRv = Sway-referenced visual support, SRs = Sway-referenced support, C = Condition, Δ (Difference) = Post–Pre.

Subsequently, the SOT sensory ratios (i.e., somatosensory, visual, vestibular and preference ratios) revealed no statistically significant training effect (Table 3).

**Table 3:** Sensory ratios calculated from SOT equilibrium scores for each group.

Group	Time-point	SOM	VIS	VES	PREF
HHS	Pre	0.949±0.066	0.800±0.196	0.641±0.186	0.961±0.046
	Post	0.926±0.093	0.793±0.173	0.640±0.110	0.970±0.072
	Δ	-0.023±0.027	-0.007±0.023	-0.001±0.080	0.009±0.026
VHS	Pre	0.964±0.019	0.780±0.131	0.564±0.094	0.974±0.030
	Post	0.960±0.033	0.815±0.157	0.611±0.108	0.971±0.040
	Δ	0.004±0.014	0.035±0.026	0.047±0.014	0.003±0.010
NHS	Pre	0.948±0.030	0.872±0.141	0.731±0.087	0.961±0.046
	Post	0.936±0.051	0.805±0.180	0.714±0.140	0.962±0.051
	Δ	-0.012±0.021	-0.067±0.039	-0.017±0.053	0.001±0.031
CTL	Pre	0.966±0.027	0.870±0.103	0.686±0.096	0.965±0.044
	Post	0.985±0.017	0.888±0.074	0.741±0.082	0.960±0.029
	Δ	0.019±0.010	0.018±0.029	0.055±0.014	-0.005±0.015

HHS = Horizontal headshake, VHS = Vertical headshake, NHS = No headshake, CTL = Control, SOM = Somatosensory ratio, VIS = Visual ration, VES = Vestibular ratio, PREF = Preference ratio, Δ (Difference) = Post–Pre.

**Fig 3:**
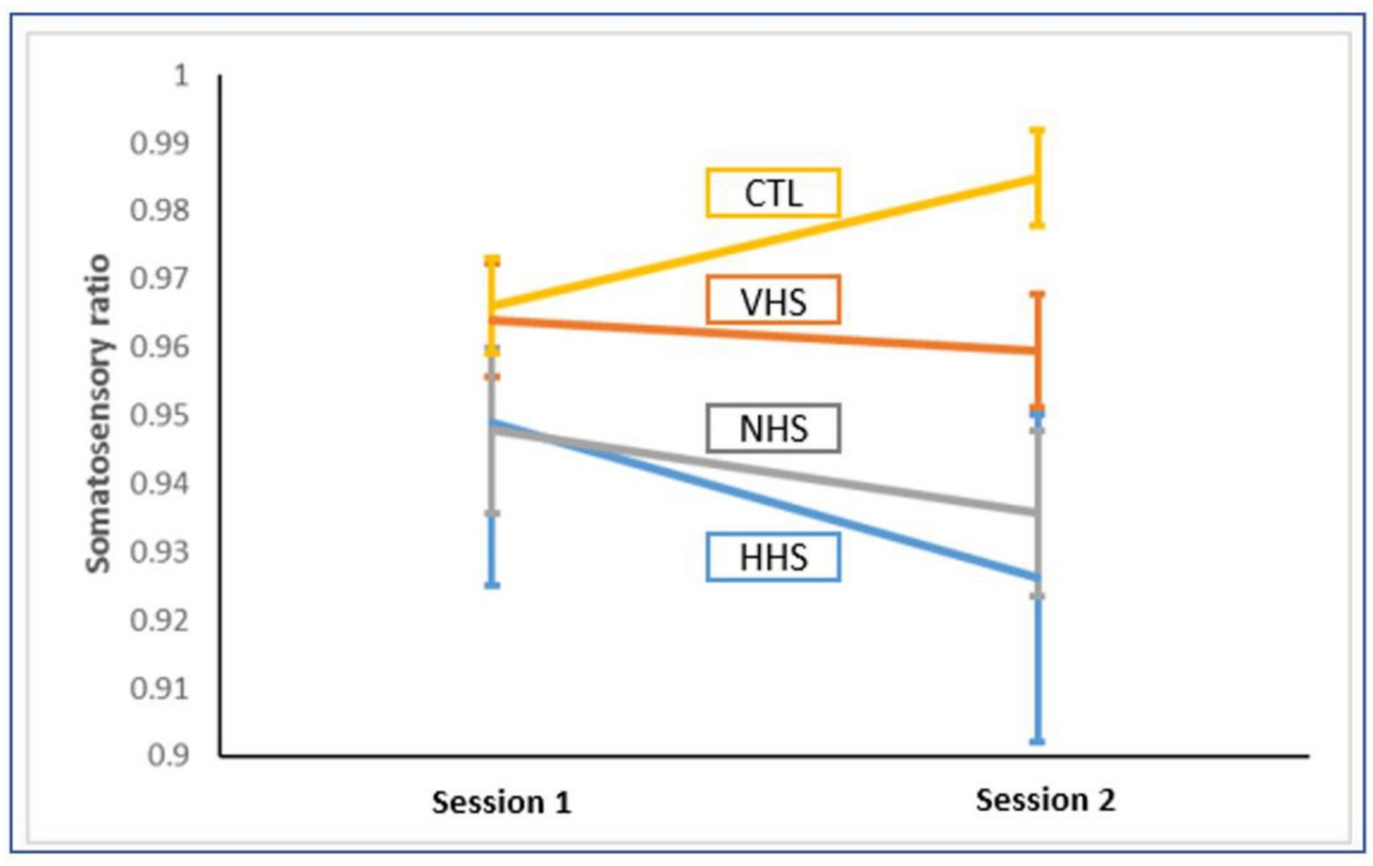
Somatosensory ratio pre- vs post-training. All three training groups showed a decreased somatosensory ratio, with the controls showing an increase. Headshake groups (HHS+VHS) showed a significantly decreased somatosensory ratio compared to CTL but not to NHS. Vertical bars indicate standard error bars.

However, there was a trending group x session interaction in the somatosensory ratio (*F*_3,38_ = 2.66, *p* = .062). All three training groups showed downweighting of SOT sensory ratio with HHS and NHS demonstrating the biggest downweighting, followed by VHS (Fig 3).

When both headshake groups were pooled together (HHS+VHS), a statistically significant group x session interaction (*F*_2,39_ = 3.24, *p* = .050) was found when compared to NHS and CTL groups separately. It demonstrated a statistically significant difference between the headshake groups and controls (*F*_1,29_ = 5.59, *p* = .025), and between NHS and controls (*F*_1,19_ = 5.97, *p* = .024). There were no differences between the headshake groups and NHS (*F*_1,30_ = 0.02, *p* = .889), which allowed us to pool all training groups together (i.e., HHS+VHS+NHS) and compare to the control group, which revealed a significant group x session interaction (*F*_1,40_ = 6.61, *p* = .014).

### Dynamic postural measures

There was a statistically significant group x session interaction due to training in the center of pressure (COP) sway velocity Std (*F*_3,37_ = 4.35, *p* = .010; average toes up trials) as assessed by the first-200ms following toes up perturbation trials (Table 4).

**Table 4:** COP of toes up perturbation trials in the first-200ms prior to the ramp start (P-values for group- by-session interactions).

COP Variable	Trial			
	1	2	3	Average
Sway area	0.280	0.466	**0.039**	**0.063**
Velocity Std	0.449	0.171	**0.075**	*0*.010*
ML Std	0.192	0.733	**0.049**	0.117

Std = standard deviation (variability), COP = center of pressure, significant values in bold and asterisks, trending values in bold only. Difference calculated by Post–Pre. *P* ≤ 0.017 for individual trials, *p* ≤ 0.05 for averaged trials.

**Fig 4:**
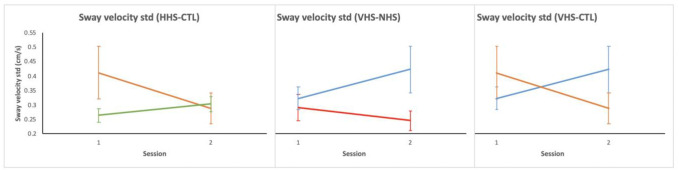
Planned comparison of groups for COP sway velocity Std during toes up trials. Green = HHS, blue = VHS, red = NHS, orange = CTL. Vertical bars indicate standard error bars. Headshake groups (HHS & VHS) showed increased sway velocity variability compared to NHS and CTL groups.

Planned comparison of the sway velocity Std (i.e., sway velocity variability) showed that the differences were between HHS and CTL (0.038 cm/s vs -0.123 cm/s; *F*_1,18_ = 7.11, *p* = .016), VHS and NHS (0.100 cm/s vs -0.046 cm/s; *F*_1,19_ = 4.66, *p* = .044), and VHS and CTL (0.100 cm/s vs -0.123 cm/s; *F*_1,17_ = 6.95, *p* = .017) (Fig 4).

**Fig 5:**
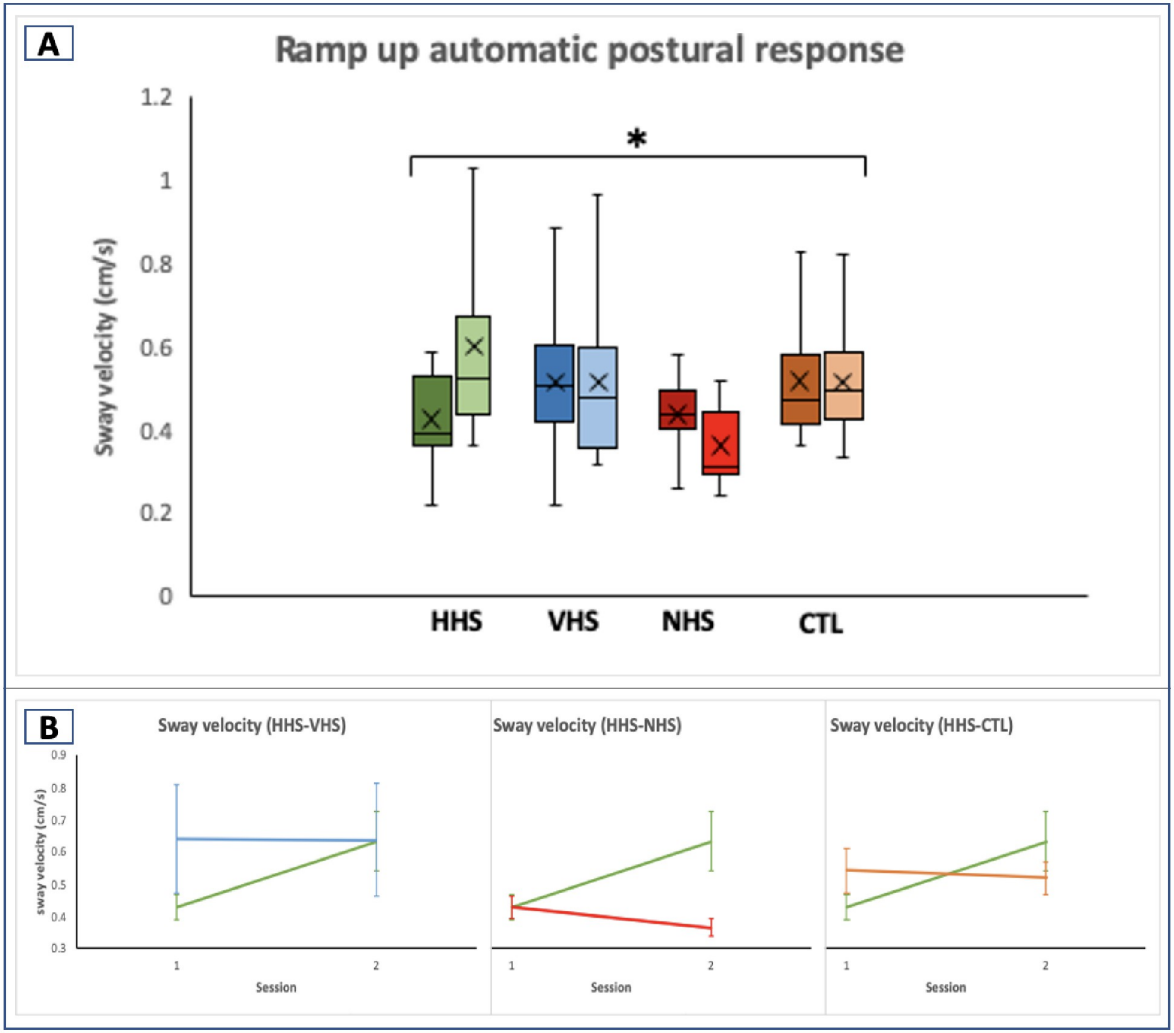
COP sway velocity pre- and post-training. A. Automatic postural balance during toes up perturbation trials for the 4 groups. Lighter colors indicate post-assessment. HHS group showed increased sway velocity, while NHS group showed a decreased sway velocity and no change in VHS and CTL groups. B. Planned comparison of 3 groups compared to HHS. Green = HHS, blue = VHS, red = NHS, orange = CTL. Vertical bars indicate standard error bars. HHS increased in sway velocity compared to all the other groups. * indicates significance at *p* ≤ 0.05.

Also, there was a statistically significant group x session interaction due to training in the COP sway velocity for automatic postural responses for the average toes up trials (*F*_3,37_ = 5.60, *p* = .003) (Fig 5A). Planned comparison showed that the change in velocity was found between HHS and VHS (0.205 cm/s vs -0.002 cm/s; *F*_1,19_ = 5.27, *p* = .033), HHS and NHS (0.205 cm/s vs -0.065 cm/s; *F*_1,20_ = 9.16, *p* = .007), and HHS and control (0.205 cm/s vs -0.024 cm/s; *F*_1,18_ = 5.47, *p* = .031) (Fig 5B). There were no significant group x session interactions in the toes down trials.

### Eye movement variables

A statistically significant group x session interaction in the horizontal eye movement for the mean toes down trials was found (*F*_3,30_ = 3.65, *p* = .024) in the pre- and post-training (Table 5).

**Table 5:** P-values of eye movement change score variables for toes down trials (1^st^– 200ms after ramp rotation onset); p-values show session-by-group training effect.

EOG Variable	Trial			
	1	2	3	Average
EM velocity (deg/s)				
HHS	1.10±7.71	-4.16±7.48	-1.08±2.71	-1.22±5.50
VHS	-1.68±15.29	4.23±10.0	0.03±2.24	0.66±6.28
NHS	1.34±6.46	-1.53±2.88	-0.27±3.54	-0.29±3.60
CTL	-2.88±8.13	0.98±2.94	0.14±1.97	-0.59±3.16
***p-value***	0.802	**0.036**	0.610	0.826
Horizontal EM (deg)				
HHS	-0.55±1.43	-0.20±0.68	-0.50±0.90	-0.37±0.64
VHS	0.17±0.86	0.82±1.54	0.37±0.59	0.41±0.65
NHS	0.23±0.48	-0.33±1.12	-0.69±1.75	-0.26±1.02
CTL	-0.07±0.70	0.32±0.49	0.06±0.78	0.10±0.48
***p-value***	0.413	**0.081**	0.153	*0*.024*

EM = eye movement, Significant values in bold, italics and asterisks; trending values in bold only. *p* ≤ 0.017 for individual trials, *p* ≤ 0.05 for averaged trials.

**Fig 6:**
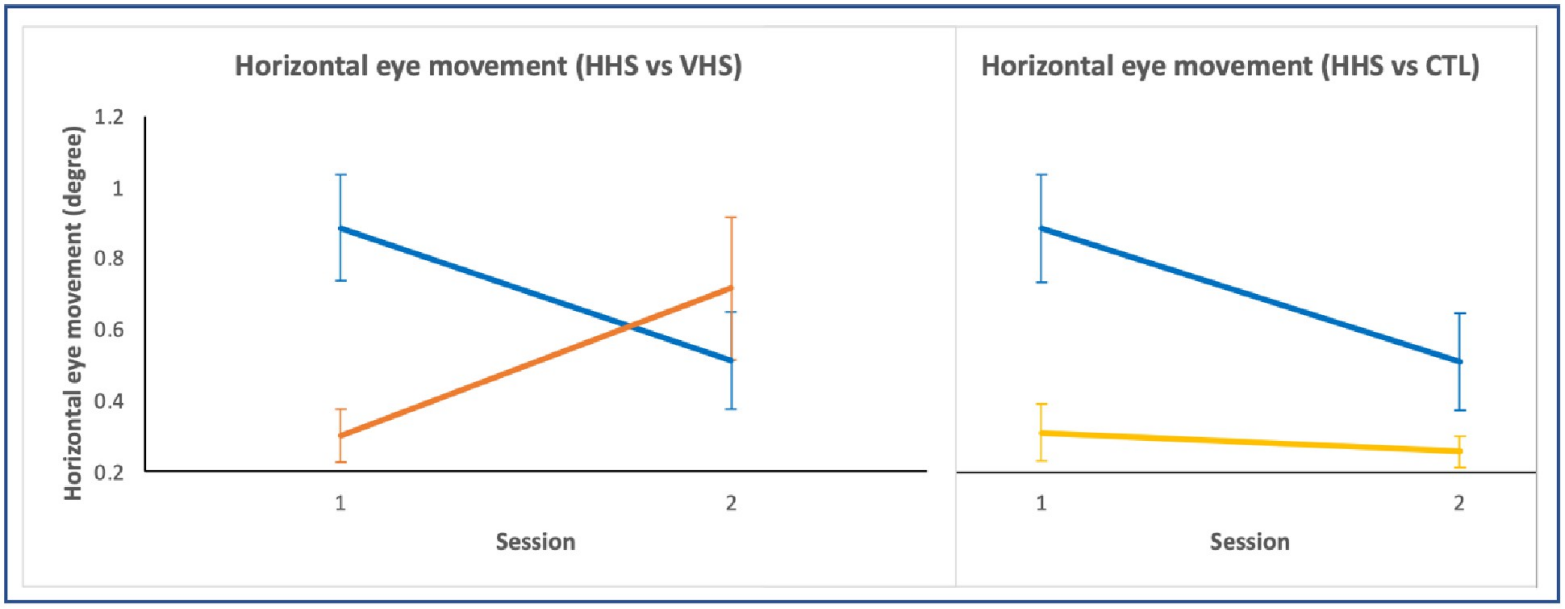
Planned comparison of groups for horizontal eye movement during toes down trials. Blue = HHS, orange = VHS, yellow = CTL. Vertical bars indicate standard error bars. HHS showed decreased horizontal eye movement compared to VHS and CTL.

Planned comparison showed changes between HHS and VHS (*F*_1,17_ = 9.00, *p* = .008), and HHS and control (*F*_1,18_ = 4.57, *p* = .046) with a decrease in horizontal eye movement in HHS (Fig 6).

There were no statistically significant differences in the other eye movement variables.

## Discussion

Our study examined whether concurrent headshake activity and weight-shift training would affect vestibular recalibration and sensory reweighting of postural control. Rhythmic headshake activity coupled with WST demonstrated a significant decrease in horizontal eye movement in the HHS group. There was also a significantly faster automatic postural response (for HHS) and improved flexibility (for both headshake groups) following the training. Furthermore, there were changes in two other variables in the headshake groups; decreased horizontal VOR gain and somatosensory reweighting were found when comparing the pooled headshake data (HHS+VHS) to the no-headshake groups (NHS and control). The findings suggest that headshake activity concurrent with postural training recalibrates the vestibular system [19] through adaptation, which stabilizes gaze and influences postural control.

### Horizontal vestibular activation affects gaze stabilization by decreasing horizontal VOR gain and horizontal eye movement

The headshake activities coupled with WST rely on several processes. Voluntary saccades and VOR help stabilize eye movements [32, 33], while vestibulo-collic (VCR) and cervico-collic (CCR) reflexes together with somatosensory input are needed to appropriately control head movements and weight shifts on different surfaces in order to maintain postural control [20, 21]. The outcome of the present study showed a decrease in the horizontal VOR gain which contributed to the decrease in horizontal eye movement. These two different measurements complement each other and provide evidence that our intervention can lead to gaze stabilization. Gaze stabilization occurs as a result of changing the relationship between the motor output and sensory input of the head [34] as they are coordinated with voluntary saccadic and fixation activity. In our protocol, not only was vestibular-mediated oculomotor control trained, but also voluntary saccades were required to drive the eyes quickly from one target to the next, which were cued every 4s. The voluntary head shaking movements combined with frequent visual target shifts required well-calibrated gaze stabilization in order to foveate the target. Clinically, saccades can be used to replace the slow phase of the VOR component to correct abnormal eye movements and stabilize gaze [18]. Compared to vertical headshake (VHS), the horizontal headshake activity clearly led to a reduction in horizontal eye movement during the subsequent (i.e., post-assessment) fast toes down perturbations resulting in better fixation during the task. Horizontal HS activity predominantly stimulates the horizontal semicircular canals (SCCs) [35], and activates horizontal VOR, while vertical HS involves a complex interaction of anterior and posterior SCCs, otoliths, with vertical VOR, VCR, and CCR. We can only speculate that the differences in these tasks require different amounts of training to cause sustained, measurable adaptation in eye movement activity, however, the current training protocols did induce significant changes in vestibular and oculomotor activity during subsequent postural challenges.

Decreased VOR gain particularly in the headshake groups is indicative of vestibular reflex recalibration, which may be carried out through the simultaneous enhancement of vestibular signals via cerebellar long-term potentiation (LTP) and suppression of eye movement signals via long-term depression (LTD) [36]. The rhythmic headshake activity coupled with the WST may have influenced these long-term processes known to play a role in vestibular adaptation, thus attenuating VOR gain [36]. Apart from vestibular adaptation, the decreased VOR gain mediated by the LTP and LTD processes could also be related to the adaptive learning that occurs during vestibular habituation. During habituation, the excitatory postsynaptic potential (EPSP) is gradually reduced in amplitude which can lead to temporary reduction of dizziness. Moreover, a permanent decrease in symptoms such as dizziness may occur after further repeated exposures to the provocative movements, whereby synaptic connections decrease in number leading to long-term structural changes [4, 37]. While this could be a process of interest in individuals who suffer from vestibular and/or dizziness issues, in the healthy young population tested in the current study, exercises were well tolerated. Therefore, symptoms of dizziness, headache and nausea were rarely present, and showed only marginal changes. Due to this limitation, in future studies, neurologically impaired patients should be investigated to determine if an association exists between vestibular symptoms and VOR gain, which would help determine if vestibular habituation is a significant mechanism in concurrent HS-WST.

### Sensory reweighting may occur following vestibular-postural training

The SOT composite score and sensory ratios did not yield a significant difference possibly due to a ceiling effect in an otherwise healthy population, however, a secondary analysis did reveal somatosensory downweighting for the pooled headshake groups (i.e., HHS+VHS vs. NHS vs. controls). This somatosensory downweighting was contrary to our hypothesis and the results in our previous study [17]. It was hypothesized in the current study that headshake activity would downweight the vestibular system and in turn drive compensatory upweighting of the somatosensory and/or visual channels. However, the observed somatosensory downweighting suggests that this channel is less likely to be driven by the headshake activity alone (as seen in both headshake groups), rather it may have also been under the influence of the repetitive sensorimotor task of WST (as seen in the NHS group). As the larger somatosensory downweighting was found in the headshake groups it suggests that there could be a central integration of both vestibular and somatosensory inputs from the headshake activity and the WST. Since this study did not have a headshake group with no WST, we cannot clearly determine the strength of influence of the WST versus vestibular activation on somatosensory reweighting. However, the magnitude of the change in somatosensory reweighting, VOR gain and the significant change in horizontal eye movement was larger for the HHS group. This suggests that the concurrent horizontal vestibular activation and WST produces a larger training effect compared to WST alone.

### Vestibular activation improves postural flexibility and automatic postural response

The toes up trials resulted in significant differences in the average COP sway velocity standard deviation (i.e., sway variability). Similar to our findings, Chen and colleagues [28] found toes up perturbations to induce larger and faster medio-lateral center of mass (COM) than toes down perturbation. Vestibular participants compared to age-matched controls have also demonstrated greater instability in trunk roll (i.e., ML variability) compared to pitch movements [38]. The results of our study support the evidence that vestibular training can be evaluated by employing sway variables during toes up perturbation trials.

Compared to the other groups (i.e., NHS and control), the vestibular activation groups (i.e., HHS and VHS), particularly the VHS group, showed an increased sway velocity variability in postural control following the training. More variability could indicate better flexibility and larger limits of stability for balance control. For instance, participants with Parkinson’s disease (PD) may exhibit lower variability compared to age-matched healthy participants, which generally does not correlate with better postural control in the PD population. The lower variability in the PD may be due to rigidity and inflexibility making the patients prone to falls [39]. The important factor is not variability per se, but the sway measure being within the optimal range. Additionally, since the velocity variability for NHS and control decreased after training, it is suggestive of postural inflexibility thus lowering postural stability. Furthermore, automatic postural response showed a difference in postural control following training between the headshake groups. This outcome measure as expressed by the sway velocity increased for HHS, indicating a faster automatic (or reflexive) response. Meanwhile, the sway velocity in the VHS and control did not change and participants in the NHS responded slower. These findings suggest that horizontal headshake produces a vestibular adaption effect through the recalibration of VCR and VSR, resulting in a faster reflex response.

### Limitations and further studies

One limitation is that participants were healthy young adults without vestibular impairment. It may be more difficult to induce large changes in individuals with a healthy system, and mechanisms for change may be different in individuals with vestibular deficits. In previous studies with healthy dancers that showed vestibular adaptation, the amount of training of participants was on average 16.6 h/week for 9.2 years [40]. The 20 mins of training/day for only five sessions tested in our study was relatively short to expect large vestibular changes. Typically, 6–8 weeks of training would be used to show larger changes between the groups. In addition, a follow-up was not performed to ascertain whether the gains would lead to long-term retention.

## Conclusion

Concurrent vestibular activation and WST demonstrated significantly decreased horizontal eye movement and increased automatic postural response in the horizontal headshake group. The study also revealed significantly decreased VOR gain and somatosensory downweighting coupled with improved postural flexibility in the training groups with headshake. The current findings suggest there is a predominant influence of vestibular recalibration supporting adaptation and probable habituation through headshaking and sensory reweighting. The findings may also serve as a basis for the development of a treatment regimen for impaired neurological populations, such as those with vestibular disorders or sensory integration problems as seen in traumatic brain injury, stroke and the elderly under the supervision of a rehabilitation specialist.

## Supporting information

S1 Checklist Human participants research checklist. (DOCX)

## References

[1] Nashner L, Berthoz A. Visual contribution to rapid motor responses during postural control. Brain Research. 1978;150(2):403–7. doi: 10.1016/0006-8993(78)90291-3 678978

[2] Nashner LM. Adaptation of human movement to altered environments. Trends in Neurosciences. 1982;5(C):358–61.

[3] Guskiewicz KM. Postural stability assessment following concussion: One piece of the puzzle. Clinical Journal of Sport Medicine. 2001;11(3):182–9. doi: 10.1097/00042752-200107000-00009 11495323

[4] Shumway-Cook A., & Woollacott MH. Motor control: translating research into clinical practice. Lippincott Williams & Wilkins; 2007.

[5] Peterka RJ. Sensorimotor integration in human postural control. Journal of neurophysiology. 2002;88(3):1097–118. doi: 10.1152/jn.2002.88.3.1097 12205132

[6] Black FO, Pesznecker SC. Vestibular adaptation and rehabilitation. Current Opinion in Otolaryngology and Head and Neck Surgery. 2003;11(5):355–60. doi: 10.1097/00020840-200310000-00008 14502066

[7] Hall CD, Herdman SJ, Whitney SL, Cass SP, Clendaniel RA, Fife TD, et al. Vestibular Rehabilitation for Peripheral Vestibular Hypofunction. Vol. 40, Journal of Neurologic Physical Therapy. 2016. 124–155 p. 26913496 10.1097/NPT.0000000000000120 PMC4795094

[8] McDonnell MN, Hillier SL. Vestibular rehabilitation for unilateral peripheral vestibular dysfunction. Cochrane Database of Systematic Reviews. 2015;36(3):248–9. doi: 10.1002/14651858.CD005397.pub4 25581507 PMC11259236

[9] Herdman S. Vestibular rehabilitation. Current Opinion in Neurology. 2013;26(1):96–101. doi: 10.1097/WCO.0b013e32835c5ec4 23241567

[10] Ricci NA, Aratani MC, Doná F, Macedo C, Caovilla HH, Ganança FF. Revisão sistemática sobre os efeitos da reabilitação vestibular em adultos de meia-idade e idosos. Rev bras fisioter. 2010 Oct;14(5):361–71. 21180862

[11] Mitsutake T, Sakamoto M, Ueta K, Oka S, Horikawa E. Effects of vestibular rehabilitation on gait performance in poststroke patients. International Journal of Rehabilitation Research. 2017;40(3):240–5. 28542112 10.1097/MRR.0000000000000234

[12] Telian SA, Shepard NT, Smith-Wheelock M, Kemink JL. Habituation Therapy for Chronic Vestibular Dysfunction: Preliminary Results. Otolaryngol Head Neck Surg. 1990 Jul;103(1):89–95. doi: 10.1177/019459989010300113 2117736

[13] Clendaniel RA. The Effects of Habituation and Gaze Stability Exercises in the Treatment of Unilateral Vestibular Hypofunction: A Preliminary Results. Journal of Neurologic Physical Therapy. 2010 Jun;34(2):111–6. doi: 10.1097/NPT.0b013e3181deca01 20588098 PMC2904475

[14] Writer H, Arora R. Vestibular Rehabilitation: An Overview. An International Journal of Otorhinolaryngology Clinics. 2012 Apr;4(1):54–69.

[15] Morimoto H., Asai Y., Johnson E. G., Lohman E. B., Khoo K., Mizutani Y., et al. Effect of oculo-motor and gaze stability exercises on postural stability and dynamic visual acuity in healthy young adults. Gait & posture. 2011;33(4):600–3. doi: 10.1016/j.gaitpost.2011.01.016 21334899

[16] Matsugi A., Ueta Y., Oku K., Okuno K., Tamaru Y., Nomura S., et al. Effect of gaze-stabilization exercises on vestibular function during postural control. Neuroreport. 2017;28(8):439–43. doi: 10.1097/WNR.0000000000000776 28368883

[17] Appiah-Kubi KO, Wright WG. Vestibular training promotes adaptation of multisensory integration in postural control. Gait & Posture. 2019;73(July):215–20. doi: 10.1016/j.gaitpost.2019.07.197 31376748

[18] Aligene K, Lin E. Vestibular and balance treatment of the concussed athlete. Greenwald BD, Gurley JM, editors. NeuroRehabilitation. 2013 May 21;32(3):543–53. doi: 10.3233/NRE-130876 23648608

[19] Toth AJ, Harris LR, Zettel J, Bent LR. Vision can recalibrate the vestibular reafference signal used to re-establish postural equilibrium following a platform perturbation. Exp Brain Res. 2017 Feb;235(2):407–14. doi: 10.1007/s00221-016-4801-7 27752729

[20] Herdman SJ. Vestibular Rehabilitation. Current opinion in neurology. 2007;26(1):608. 10.1097/WCO.0b013e32835c5ec4 23241567

[21] Yates BJ, McCall AA. Compensation following bilateral vestibular damage. Frontiers in Neurology. 2011;2(88):1–13. doi: 10.3389/fneur.2011.00088 22207864 PMC3246292

[22] Faul F, Erdfelder E, Lang AG, Buchner A. G*Power 3: A flexible statistical power analysis program for the social, behavioral, and biomedical sciences. In: Behavior Research Methods. 2007. 10.3758/bf03193146 17695343

[23] Sealed Envelope Ltd. Simple randomisation service [Internet]. 2022 [cited 2023 Dec 17]. https://www.sealedenvelope.com/simple-randomiser/v1/

[24] MacDougall HG, Weber KP, McGarvie LA, Halmagyi GM, Curthoys IS. The video head impulse test: Diagnostic accuracy in peripheral vestibulopathy. Neurology. 2009;73(14):1134–41. doi: 10.1212/WNL.0b013e3181bacf85 19805730 PMC2890997

[25] McGarvie LA, MacDougall HG, Halmagyi GM, Burgess AM, Weber KP, Curthoys IS. The video head impulse test (vHIT) of semicircular canal function—age-dependent normative values of VOR gain in healthy subjects. Frontiers in Neurology. 2015;6(JUL):1–11. doi: 10.3389/fneur.2015.00154 26217301 PMC4495346

[26] Nashner LM, Peters JF. Dynamic posturography in the diagnosis and management of dizziness and balance disorders. Neurologic clinics. 1990; 2193215

[27] Coogler C. E., Catlin P. A., Bruno L., French K., & Kelly S. Consistency of human postural responses as measured by EquiTest. Posture and Gait: Control Mechanisms. Portland, Ore: University of Oregon Books; 1992. 361–364 p.

[28] Chen CL, Lou SZ, Wu HW, Wu SK, Yeung KT, Su FC. Effects of the type and direction of support surface perturbation on postural responses. Journal of NeuroEngineering and Rehabilitation. 2014 Dec;11(1):1–12. doi: 10.1186/1743-0003-11-50 24708582 PMC3986462

[29] Mahon A, Bendžiūtė S, Hesse C, Hunt AR. Shared attention for action selection and action monitoring in goal-directed reaching. Psychological Research. 2020;84(2):313–26. doi: 10.1007/s00426-018-1064-x 30097712 PMC7040085

[30] Robins RK, Hollands MA. The effects of constraining vision and eye movements on whole-body coordination during standing turns. Experimental Brain Research. 2017;235(12):3593–603. doi: 10.1007/s00221-017-5079-0 28884336

[31] Ross AI, Schenk T, Hesse C. The effect of gaze position on reaching movements in an obstacle avoidance task. PLoS ONE. 2015;10(12):1–16. 10.1371/journal.pone.0144193 PMC4670101 26636966

[32] Fischer B, Ramsperger E. Human express saccades: extremely short reaction times of goal directed eye movements. Experimental Brain Research. 1984;57(1):191–5. doi: 10.1007/BF00231145 6519226

[33] Della Santina CC, Cremer PD, Carey JP, Minor LB. Comparison of Head Thrust Test With Head Autorotation Test Reveals That the Vestibulo-ocular Reflex Is Enhanced During Voluntary Head Movements. Arch Otolaryngol Head Neck Surg. 2002 Sep 1;128(9):1044. doi: 10.1001/archotol.128.9.1044 12220209

[34] Maas EF, Huebner WP, Seidman SH, Leigh RJ. Behavior of human horizontal vestibulo-ocular reflex in response to high-acceleration stimuli. Brain Research. 1989 Oct;499(1):153–6. doi: 10.1016/0006-8993(89)91145-1 2804663

[35] Spoor F, Wood B, Zonneveld F. Implications of early hominid labyrinthine morphology for evolution of human bipedal locomotion. Nature. 1994;369(6482):645–8. doi: 10.1038/369645a0 8208290

[36] Inagaki K., & Hirata Y. Computational theory underlying acute vestibulo-ocular reflex motor learning with cerebellar long-term depression and long-term potentiation. Cerebellum. 2017;16(4):827–39. doi: 10.1007/s12311-017-0857-6 28444617

[37] Tee L, Chee N. Vestibular Rehabilitation Therapy for the Dizzy Patient. Ann Acad Med Singapore. 2005;34(4):289–94. 15937569

[38] Allum JHJ, Oude Nijhuis LB, Carpenter MG. Differences in coding provided by proprioceptive and vestibular sensory signals may contribute to lateral instability in vestibular loss subjects. Experimental Brain Research. 2008;184(3):391–410. doi: 10.1007/s00221-007-1112-z 17849108

[39] Horak FB, Nutt JG, Nashner LM. Postural inflexibility in parkinsonian subjects. Journal of the Neurological Sciences. 1992;111(1):46–58. doi: 10.1016/0022-510x(92)90111-w 1402997

[40] Maheu M, Behtani L, Nooristani M, Delcenserie A, Champoux F. Enhanced vestibulo-ocular reflex suppression in dancers during passive high-velocity head impulses. Experimental Brain Research. 2019;237(2):411–6. doi: 10.1007/s00221-018-5431-z 30426147

